# Amyotrophic Monoplegia Secondary to Posttraumatic Rupture of the Brachial Plexus's Roots: A Case Report and Review of the Literature

**DOI:** 10.1155/2021/6614881

**Published:** 2021-03-16

**Authors:** Oumniya Abouhanine, Hasnaa Belgadir, Vianney Ndayishimiye, Omar Amriss, Aicha Merzem, Nadia Moussali, Naima El Benna

**Affiliations:** Department of Radiology, Ibn Rochd University Hospital Center of Casablanca, Radiology Service of August 20, 1953 Hospital, Hassan II University of Casablanca, Morocco

## Abstract

Brachial plexus lesions most often occur in multiple trauma. We report a case of a 37-year-old patient who presented an upper left limb total sensitivomotor deficit and amyotrophy after a cervical and upper limb trauma. Cervical magnetic resonance imaging (MRI) was performed. It noted pseudomeningoceles at the levels of C6-C7, C7-D1, and D1-D2 in T1 hyposignal , T2 and STIR hypersignal , not enhanced by the injection of Gadolinium extending in foraminal and extraforaminal spaces without visualization of the corresponding rootlets. Traumatic brachial plexus injury is a potentially serious debilitating injury which can be well explored on MRI.

## 1. Introduction

Brachial plexus injury is the most serious nerve damage to the extremities causing functional impairment of the upper limb. It is usually the result of blunt trauma with considerable traction on the shoulder.

Our goal is to describe radiological elements pointing a posttraumatic rupture of the brachial plexus in magnetic resonance imaging.

## 2. Case Presentation

We report a case of a 37-year-old patient who presented an upper left limb total sensitivomotor deficit and amyotrophy after a cervical and upper limb trauma occurred 7 years ago treated in a peripheral hospital, referred to our service for cervical MRI exploration.

The protocol used associated T1, T2, and STIR sagittal sequences, T2 axial sequences, and T1 sagittal and axial sequences after injection of Gadolinium.

**Figure 1:**
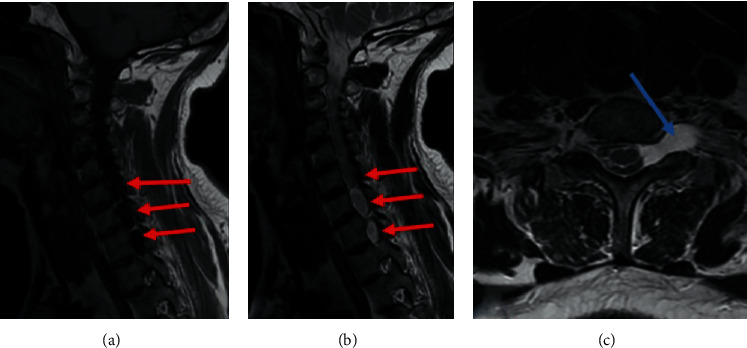
Cervical MRI, (a, b) T1 and T2 sagittal and (c) T2 axial sequences: pseudomeningoceles in the form of oblong formations of the C6-C7, C7-D1, and D1-D2 levels (red arrows) in T1 hyposignal, T2 hypersignal, extending into foraminal, and extraforaminal spaces (blue arrow). Note also the nonvisualization of the anterior and posterior rootlets, better visible on sagittal sections in favor of total avulsion.

**Figure 2:**
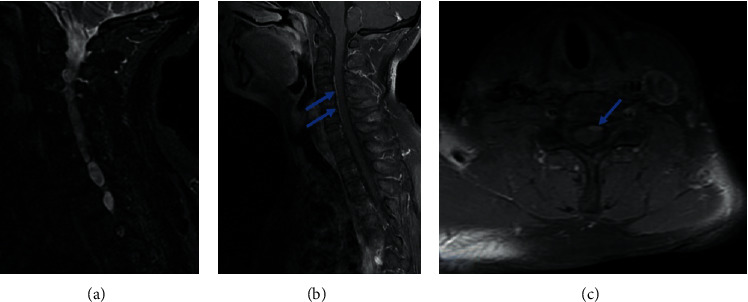
Cervical MRI, (a) STIR sagittal and (b, c) T1 sagittal and axial sequences after injection of Gadolinium; nodular contrast enhancement on the anterior surface of the left hemimarrow (blue arrows): appearance in favor of preganglionic lesions.

It noted pseudomeningoceles at the levels of C6-C7, C7-D1, and D1-D2 in T1 hyposignal, T2 and STIR hypersignal (Figure 1), not enhanced by the injection of Gadolinium extending in foraminal and extraforaminal spaces without visualization of the corresponding rootlets. There was a nodular contrast enhancement on the anterior surface of the left hemimarrow (Figure 2).

## 3. Discussion

Traumatic lesions of the brachial plexus mainly affect young individuals. According to the main studies reported in the literature, motorcycle crashes are the most common cause of such injuries [1].

Traumatic injury to the brachial plexus is secondary to direct stretching injuries, bruises, or wounds aggravated by hematoma and the presence of foreign bodies.

Traumatic lesions of the brachial plexus are classically divided into preganglionic lesions (complete or partial root avulsions) and postganglionic lesions corresponding to damage to the trunks or bundles, which may be or not reversible. Concomitant pre- and postlymph node involvement is observed in 15% of cases [2].

Clinically, manifestations are correlated with the injury level, such as active and passive movements of the shoulder, forearm, arm, hand, and wrist, across a full range of motion. Muscle classification systems such as LSUHSC (Louisiana State University Health Science Center) grades and MRC (Medical Research Council) grades have been used to assess muscle power. Injury to the upper brachial plexus (C5 and C6) causes paralysis of the muscles of the shoulder and biceps. When C7 is involved, certain muscles in the wrist and forearm are affected. Injury to the inferior brachial plexus (C8 and T1) causes paralysis of the forearm flexor and intrinsic muscles of the hand [3].

At radiological exploration, MRI by associated visualization of the marrow has become the alternative to Myeloscanner, which is an invasive examination. It effectively specifies the lesional level downstream or upstream of the spinal ganglion; this distinction is essential because only lesions located below the spinal ganglion will benefit from microsurgical reconstruction. In the event of involvement of the brachial plexus, pseudomeningoceles are also very frequently found [4].

T1-weighted images are most useful for the anatomy of the nerves, surrounding fatty planes, scalene and regional muscles, and thoracic outlet. T2-weighted images can be obtained with a variety of contrasts (T2-weighted images/STIR fat-suppressed). STIR imaging has multiple advantages, such as depicting symmetrical normal hyperintensity and dorsal nerve root ganglion size characteristics at multiple levels on the same image for comparison.

The 3D STIR SPACE sequence provides excellent background fat removal and isotropic multiplanar and planar curved reconstructions. The reconstructed images can be further enhanced with MIP to highlight the imaging abnormality and for representation along the longitudinal plane of the nerve [5–7].

Ultrasound directly visualizes the following lesions:

- Nervous interruptions as well as the space between two nerve stumps
- Posttraumatic fusiform focal thickening of a nerve segment, probably due to a neuroma appearing late after the trauma
- Tissue scarring secondary to penetrating trauma [4]

## 4. Conclusion

Traumatic brachial plexus injury is a potentially serious debilitating injury. MRI is an excellent tool in the assessment of brachial plexus injury: it demonstrates the location of the nerve damage and describes nerve continuity, thus facilitating the classification of nerve damage for preoperative planning.

## Data Availability

The data are archived in the database of the radiology department of the university hospital Ibn Rochd of Casablanca.
